# Surgery of the Brainstem

**DOI:** 10.3171/2019.10.FocusVid.Intro

**Published:** 2019-10-01

**Authors:** M. Yashar S. Kalani, Robert F. Spetzler, Rudolf Fahlbusch, James K. Liu

**Affiliations:** 1 Department of Neurosurgery, University of Virginia School of Medicine, Charlottesville, Virginia;; 2 Department of Neurosurgery, Barrow Neurological Institute, Phoenix, Arizona;; 3 International Neuroscience Institute, Hannover, Germany; and; 4 Department of Neurological Surgery, Rutgers New Jersey Medical School, Newark, NJ, USA

IWe are honored to have been chosen by Drs. Couldwell and Cohen-Gadol to edit this issue of *Neurosurgical Focus: Video*, on surgery of the brainstem. Perhaps no other area in the neuroaxis is more studied, respected, and feared than the brainstem and adjacent structures. Long considered a “no-man’s-land,” surgery of the brainstem was considered a fool’s errand, with morbidity and mortality exceeding 70% until the middle of the twentieth century.

With improvements in anesthesia, the availability of neurophysiological monitoring, the introduction of advanced imaging, the operative microscope and surgical tools, and enhanced understanding of the anatomy of the brainstem, a group of daring pioneers began undertaking surgical exploration in this previously untouchable domain of the brain. The 1980s saw the development and refinement of skull base approaches. These techniques were initially maximally invasive, removing bone to minimize manipulation of the underlying structures, but were gradually refined to be less destructive. The introduction of keyhole techniques and neuroendoscopy in the 1990s allowed surgeons to minimize their footprint while approaching the brainstem. With the availability of the aforementioned adjuncts, the 2000s saw an expansion of surgery on the brainstem, led by the knowledge reported by pioneering surgeons, such as Robert Spetzler, Majid Samii, and Helmut Bertalanffy.

With a great deal about the tolerance of the brainstem to surgical manipulation having been learned, and with “safe entry zones” demarcated, approaches to the brainstem have become more common, although by no means commonplace. Surgery in this eloquent domain remains the territory of highly skilled surgeons (such as those whose videos are highlighted here), who possess the ability to properly select cases where the likelihood of success outweighs the risks of deficits, and only after a frank and detailed discussion with patients and families of the possible aftermath of surgery. Perhaps more so than any other area of the brain, surgery in and around the brainstem requires patient understanding that, more likely than not, they will have some degree of worsening of their preoperative deficits (or new deficits) that is often transient and most often, but not always, improves. Given the high rates of morbidity, surgery of the brainstem should only be offered if it improves upon the natural history of the lesion.

This video supplement contains submissions from four continents, by outstanding surgical teams with a wealth of experience operating in the brainstem. We were fortunate to have received nearly 50 high-quality videos, 22 of which were accepted for final production. These videos highlight nuances of surgical approach (such as the video by Ceccato et al.), the use of intraoperative monitoring and neuronavigation (see the video by Kalani et al.), microsurgical technique, and postoperative management for a diverse collection of diseases, including gliomas, cavernous malformations, infectious etiology, and hemangioblastomas. Videos on the finer points of surgical technique, such as that by Keles et al., remind us to prepare for the possibility of a redo surgery in this area and how to decrease the challenges associated with entering a previous operative bed. This video supplement contains videos dedicated to less common and, perhaps, controversial issues, such as resection of glioblastoma of the brainstem (see editorial by Dr. Fahlbusch) and the use of endonasal endoscopy to approach ventral lesions (see editorial by Dr. Cohen-Gadol).

It is our goal that this video supplement serve as a teaching tool for trainees and neurosurgeons interested in gaining a better understanding of the anatomy and surgical nuances for lesions in and around the brainstem. Dedicated study (ideally under the hands of a master surgeon), proper patient selection, and surgical dexterity are requirements for undertaking surgery in this domain with the expectation of good outcomes. We urge caution and restraint that surgery is not appropriate for all patients, all pathologies, and in all cases. Surgery does, however, have a role, in combination with medical treatment and radiation, for a subset of lesions and should be offered to patients as an option.

